# Evaluating the effectiveness of Community Health Worker home visits on infant health: A quasi-experimental evaluation of Home Based Newborn Care Plus in India

**DOI:** 10.7189/jogh.11.04060

**Published:** 2021-10-09

**Authors:** Thomas Alan Newton-Lewis, Girija Bahety

**Affiliations:** 1 Freelance health systems consultant, Oxford, United Kingdom; 2 Economics Department and The Fletcher School, Tufts University, USA

## Abstract

**Background:**

Home visits by community health workers are promoted to improve the coverage and uptake of evidence-based newborn services and behaviours. However, evidence on the effectiveness of these home visits delivered through government systems at scale is limited, as is evidence from the post-neonatal period. From 2013 to 2017, the Government of India piloted an intervention called Home Based Newborn Care Plus with the goal of reducing pneumonia- and diarrhoea-related morbidity and malnutrition. Village-based Accredited Social Health Activists were incentivised to make quarterly home visits to infants between three and 12 months of age. After the pilot, the intervention was adapted and scaled up nationally (with an additional visit at 15 months of age) as a new programme called Home Based Care for Young Child.

**Methods:**

The study used a quasi-experimental, difference-in-differences method to assess the quantitative impact on key outcome indicators by comparing changes over time in treatment districts with matched control districts. This was supplemented by a quantitative health worker survey and qualitative data collected at worker and community level.

**Results:**

The intervention led to a significant increase in the number of home visits, and their content became more aligned with Home Based Newborn Care Plus protocols. However, absolute levels of coverage remained low. The intervention had no detectable effect on the key outcomes of feeding practices, handwashing, iron and folic acid and oral rehydration solution supplementation, growth monitoring, and immunisation.

**Conclusions:**

Given the scale up of Home-Based Care for Young Child, there is a need to identify appropriate and comprehensive support for Accredited Social Health Activists to attain high coverage and quality and deliver impact. This will require reconsidering current design elements (such as incentives) and solving the underlying demand side and system level challenges (such as workload and supply chains) constraining Accredited Social Health Activists.

Community health workers (CHWs) are acknowledged to play a critical role in promoting the uptake of health services and the adoption of appropriate behaviours [1,2], particularly in low-resource settings such as India, which typically face health worker shortages and an increased burden on public health facilities [3,4].

Home visits after childbirth by CHWs are promoted as an evidence-based strategy to reduce newborn mortality and improve key newborn care practices and outcomes in high mortality settings [5]. For example, a systematic review found that home based newborn care is associated with a reduction in neonatal and perinatal mortality in South Asian settings with high neonatal mortality rates and poor access to health facility-based care [6]. In India, Bang et al [7] and Kumar et al [8] show strong reductions in neonatal mortality for small scale pilot projects in Gadchiroli, Maharashtra and Shivgarh, Uttar Pradesh, respectively. A cluster randomized trial across five districts in north, east, and west India also found reductions in neonatal and infant mortality [9]. In Pakistan, there is evidence of positive impacts of home visits on developmental and nutrition outcomes [10]. A recent systematic review and meta-analysis also found that CHW home visits increased early initiation of breastfeeding [11].

However, much of the evidence on the effectiveness of home visits comes from small scale pilots implemented in highly controlled field settings with the help of non-governmental organisations [1]. This study provides unique evidence on the effectiveness of home visits when implemented at scale using government systems, thus contributing to a priority research gap identified in the literature [2].

Further, whilst home visits in the neonatal period, in conjunction with other interventions, have been shown to increase care seeking for diarrhoea and pneumonia and reduce morbidity in the post-neonatal period, including in India [12], few studies provide comparable evidence on the effectiveness of home visits in the post-neonatal period or home visits focused on the prevention of morbidity rather than treatment. Home visits to children under two in Bangladesh have been shown to improve complementary feeding [13] but the overall evidence base is limited. Through its rigorous evaluation of Home Based Newborn Care Plus (hereafter, referred to as HBNC+), a large-scale pilot of a programme in which CHWs provided home visits to infants in India, this paper provides evidence on a broad scope of post-neonatal infant health outcomes.

Since 2010, CHWs in India in the form of Accredited Social Health Activists (ASHAs) have been mandated to make home visits for newborn care under a programme called Home Based Newborn Care (HBNC). ASHAs are trained female volunteers chosen from within the community, covering a population of 1000 beneficiaries [14]. In most states, ASHAs do not receive a salary and are remunerated through performance-based incentives for different tasks or services. Under HBNC, ASHAs were given financial incentives (Rs 250, approximately US$4) to undertake six home visits to the mothers of children born at health facilities (on days 3, 7, 14, 21, 28 and 42 after birth), with an additional visit on the first day to those born at home. The visits were expected to improve community newborn care practices and ensure the early detection of neonatal illnesses and appropriate referral.

From 2013 until 2017, the Government of India piloted an extension of HBNC, termed HBNC+, to focus on post-neonatal and infant health so that home visits could also be provided by ASHAs to infants up until the age of one. This was piloted in 13 districts in the four states of Bihar, Odisha, Madhya Pradesh and Rajasthan (hereafter, referred to as “treatment districts”), with a combined population of 20.6 million in 2011. After the pilot, the intervention has now been adapted and scaled up nationally (with the addition of one visit at 15 months of age) as Home Based Care for Young Child (HBYC) [15].

HBNC+ was piloted with the support of the Norway India Partnership Initiative (NIPI). The broader NIPI programme focused on supporting the Government of India’s National Health Mission (NHM) to pilot continuum of care innovations at the community and facility level [16].

The HBNC+ intervention packaged multiple evidence-based interventions for improving the survival and development of infants through additional incentivised, structured home visits by ASHAs at three, six, nine, and 12 months of age. HBNC+ targeted the prevention of pneumonia- and diarrhoea-related morbidity and malnutrition. As such, it did not focus on the assessment, classification, referral and treatment of sick infants. The home visits were expected to promote exclusive breastfeeding for the first six months after childbirth, ensure continued breastfeeding and complementary feeding from six months, promote routine child immunisation, provide counselling for handwashing, facilitate the distribution of oral rehydration solution (ORS) and iron and folic acid (IFA) syrup, ensure regular growth monitoring, and promote Early Child Care and Development (ECCD) [17].

Under the existing NHM protocol, ASHAs were already expected to make monthly home visits to households with infants. The additionality of HBNC+ was a focused push to ensure that home visits happened at key moments of a child’s growth with key target messages. This was to be achieved through the training of ASHAs in the HBNC+ protocol and by the provision of incentives (Rs 250 per child, approximately US$4) to ASHAs for the successful completion of four visits, as per the defined schedule. For a typical ASHA’s catchment area in the programme states, HBNC+ would require ten home visits per month, on top of the sixteen required for HBNC (authors’ calculations).

## METHODS

The effectiveness of HBNC+ was evaluated using a mixed methods approach, combining both quantitative and qualitative methods. This paper presents a subset of the broader evaluation undertaken by Oxford Policy Management and Sambodhi Research and Communications [18]. The evaluation used a quasi-experimental approach, comparing changes in target outcomes in treatment districts that received the HBNC+ intervention with changes in matched control areas. The evaluation terminology is based on the common framework developed by Bryce et al. [19].

The evaluation was structured around two core research questions at the population level:

1. Coverage and quality: Did the programme inputs and processes (training and incentives) lead to additional outputs in the form of home visits by ASHAs to target households according to the HBNC+ protocol?
2. Outcomes: Did the home visits lead to improved preventive health practices (handwashing, exclusive and continued breastfeeding, and complementary feeding), improved interaction with the child (through ECCD), increased use of products (ORS and iron supplementation), and service uptake (growth monitoring and full immunisation)?

Although the final goal of the programme was to reduce disease morbidity and infant mortality, the evaluability of these impact indicators was constrained by long transmission mechanisms, small effect sizes, and overarching confounding factors. Therefore, the evaluation investigated the impact of the programme only as far as the output and the outcome level (service uptake and health behaviours) of the results chain.

The quantitative impact evaluation involved primary data collection at the population and health worker level, and the qualitative impact evaluation involved data collected through focus group discussions (FGDs) with mothers and in-depth interviews (IDIs) with ASHAs. Fieldwork was undertaken in December 2013-January 2014 (baseline) and January-February 2017 (endline). Data was collected by Sambodhi Research and Communications.

### Quantitative evaluation at population level

**Figure 1:**
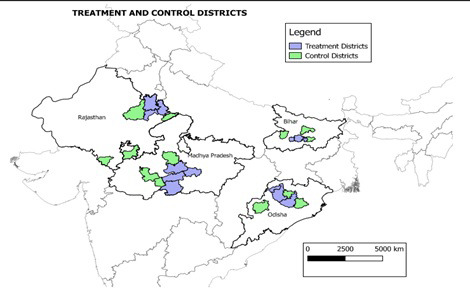
Treatment and control districts.
- Step 2: From each treatment district, two sub-districts were randomly sampled and matched with two sub-districts from the matched control district, based on the factors listed above (excluding population density), using the minimum difference approach [20]. For each treatment sub-district, the sub-district in the control district with the minimum difference across indicators was chosen as the matched district, ensuring a unique match.
- Step 3: From each sampled sub-district, six PSUs were selected using the probability proportional-to-size (PPS) method. PPS is a simple sampling technique whereby the probability of selection of a unit is proportional to its size. PPS ensures the representativeness of the sample if the sampling units vary in size.
- Step 4: From each PSU, 15 households with mothers of children between 3 and 24 months of age were randomly selected after a listing exercise.

The quantitative evaluation at the population level was based on two cross-sectional population surveys of mothers of children between 3 and 23 months at baseline and endline, across a panel of primary sampling units (PSUs) of villages in treatment and control areas. The PSUs and mothers were selected based on the following steps:

- Step 1: Given that the programme had already selected the 13 treatment districts, control districts were chosen from a matching exercise to minimise the effect of confounding factors and create a comparable comparison group. The treatment and control districts were matched based on literacy rate, levels of urbanisation, the proportion of Scheduled Castes, and population density. Treatment and control districts are shown in **Figure 1****.**

At baseline, the total sample was 3935 children between 3 and 23 months of age (1948 in the treatment group and 1987 in the control group). At endline, the total sample was 4049 (1985 in the treatment group and 2064 in the control group). Based on the power calculations conducted at baseline, this gave a minimum detectable effect for a standard indicator of five percentage points at the programme level and ten percentage points at the state level (with the assumptions of a 50% baseline outcome level and design effect of 2.0).

The impact of HBNC+ on programme outcomes was identified using a quasi-experimental methodology of difference-in-differences (diff-in-diff). This compared changes over time in outcomes in treatment areas which received HBNC+ with changes in matched control areas where HBNC+ was not implemented. This approach removes biases arising from time-invariant, group-specific, unobserved factors. The diff-in-diff specification can be represented as follows:

*y_ijts_ = β_0_ + β_1_dT_j_ + β_2_dPost_t_ + β_3_*(*dPost_t_*)(*dT_j_*)* + β_4_X_i_ + θ_s_ + ε_ijt_*

where *y_ijts_* is the outcome of interest for individual *i* at time *t* in village *j* and state *s*. The dummy variable *dT* equals 1 if the individual is residing in a treatment village and 0 for a control village. The time dummy variable *dPost* equals 1 if the time period is 2017 (endline) and 0 for 2013 (baseline). The main parameter of interest is *β_3_*, the coefficient on the interaction term, (*dPost_t_*)(*dT_j_*). This parameter represents the intention to treat effect, measuring the average effect of the intervention on all households eligible to receive the intervention, whether or not they receive the intervention, and hence provides an unbiased estimate of effectiveness of HBNC+. *X_i_* is a vector of the individual characteristics of the mother and the household within which she resides. The choice of covariates was theory-based and included: the age and gender of her child; her own age and education level (primary, secondary, or higher); the number of previous childbirths; whether the birth took place in a health institution; the frequency of her ANC visits (whether it was between one and three visits or more than four); the age, gender, education level, religion, and caste of the household head; the number of females and the total size of the household; the household wealth index quintile category; and distance and family barriers to health care access. *θ_s_* refer to state-specific fixed effects. The standard errors are clustered at the village level. Since non-probability based matching techniques were used for identification purposes, we do not use probability weights and therefore the estimates apply to the impact estimation sample only. The unit of analysis is the individual/household.

The following limitations of the quantitative evaluation methodology should be acknowledged whilst interpreting its findings:

1. The evaluation is not powered to detect effects on morbidity or mortality; therefore, it can only measure up to the level of ‘outcomes’ in the theory of change. Some outcome estimates are also hampered by small sample sizes (eg, for the treatment of diarrhoea) and are therefore not powered to detect statistically significant changes.
2. The sampling strategy was defined to increase the likelihood of sound comparability between the treatment and control groups by allowing for the matching of districts and sub-divisions based on secondary data sources. The baseline study confirmed that there was no statistically significant difference between the treatment and control areas for the key indicators, lending robustness to the evaluation [21]. Key informant interviews and the PSU survey were used to assess whether other interventions, programmes, or contextual changes occurred unevenly in either treatment or control areas that would have violated the parallel trends assumption required for diff-in-diff to give valid estimates, but none were detected. We are therefore confident of the counterfactual validity and reliability of the diff-in-diff approach, provided that the parallel trends assumption is upheld. However, we cannot reliably test for this assumption.
3. Due to the changes to the data collection tools between baseline and endline, the diff-in-diff methodology cannot be used for some indicators. As a result, two outcome indicators rely on single difference estimates and therefore offer weaker causal attribution: (a) the ORS component of the programme was scaled up nationally, and as a result the impact analysis is pre-post covering only the treatment areas; and (b) the understanding that IFA distribution was part of the intervention was understood by the evaluation team only after the baseline data collection and, consequently, the evaluation is ex-post only, comparing treatment and control areas.
4. Outcomes related to ECCD are difficult to measure. For this paper, we used a crude indicator, constructed from a straightforward question asked of the mother about the method of interaction with her child, with options of talking/playing/listening (not mutually exclusive) to her child.
5. There are typical risks of inaccurate self-reporting of behaviours and service uptake and socially desirable answers that are inherent in health surveys at the population level.

### Quantitative evaluation at health worker level

The quantitative evaluation at the health worker level included a provider survey of ASHAs from each of the PSUs of the population survey. Each PSU represented one ASHA catchment area, so no further sampling was required. The evaluation measured their receipt of inputs, knowledge, and reported levels of service and product availability. 300 ASHAs were covered at baseline (144 treatment, 156 control) and 304 at endline (151 treatment, 153 control). This sample was not intended to achieve statistical significance.

### Qualitative evaluation

The qualitative data collected was collected at endline and has two data sources – FGDs with mothers and IDIs with ASHAs. Qualitative data was analysed through thematic framework analysis.

Twenty-six FGDs were held with mothers who had children younger than two years of age. These FGDs were held in 13 treatment districts across the four states, with two FGDs being conducted per district. FGDs explored themes such as the challenges of looking after infants; the treatment-seeking behaviour of mothers; the perception of services provided by health workers; women’s decision making; their status in the household; and their attitudes and practices related to family planning. A total of 170 women participated in the FGDs across the four states and the size of the FGDs ranged from 5 to 11 mothers.

IDIs were held with ASHAs to investigate issues such as her work schedule, roles and responsibilities, motivation and incentives, and attitudes and practices related to family planning. The data was collected from 26 ASHAs, with two ASHAs being interviewed from each of the 13 districts.

### Ethics

Ethical permission for the evaluation was granted by the independent ethics review board of the Public Healthcare Society (India). The evaluation was conducted to the highest ethical standards. This included ensuring that expectations of the participants were not raised, confidentiality was maintained, and respondents were informed about the purpose of the survey and asked to participate voluntarily. Informed verbal consent was obtained from the research subjects. Only female interviewers took the consent and interviewed the female respondents. No personal identifiers were used in any form of reporting or dissemination. Personal identifications were linked with a unique identifier and were kept securely. No information was published that could identify the respondents. Paper copies of questionnaires were stored for three years in a secure location before being destroyed; only the investigation team were able to access them. Participation in the research was voluntary and respondents were free to stop the interviews at any time or skip any questions they did not want to answer. They had the right to ask questions at any point before, during, or after the interview was completed. The research staff and the participants were informed about the purpose, methods, benefits, and intended possible uses of the research. All interviews were conducted by trained staff members and in conditions of privacy.

## RESULTS

### Coverage and quality

At endline, 95% of ASHAs had received training on HBNC+. Their level of programme awareness was very high, with 93% reporting the correct number of home visits they were expected to make, and 92% the correct schedule for these visits.

While the pilot target was for at least 80% of children to receive at least one HBNC+ visit, at endline, it was found that 69% of children in the treatment areas aged 12–23 months had received at least one visit between the ages of three and twelve months, and that only 39% received all four visits as per the schedule. Sub-group analysis shows no evidence of systematic exclusion based on caste, birth order, or wealth in treatment areas at endline (results not shown).

Given that NHM already mandated ASHAs to make general home visits during the HBNC+ period, not all the home visits recorded under HBNC+ are additional visits. As shown in **Table 1**, in the treatment areas, at baseline, 44% of mothers reported receiving at least one home visit; this increased to 69% at endline. However, in control areas, there was also an increase, from 42% to 55%. The diff-in-diff calculation shows a 10.5 percentage points (p.p.) increase in the proportion of households receiving at least one visit (statistically significant at the 1% level). Similarly, there was a 9.4 p.p. increase in the proportion of households receiving at least four visits as per protocol (statistically significant at the 1% level). The average mother in treatment areas gained 0.5 home visits because of the intervention (38.6% relative to control areas at baseline). Across the four states, the coverage of full schedule of visits varied unevenly, with Odisha reporting the highest coverage at 70% in treatment areas to Rajasthan reporting 18% (results not shown).

**Table 1:** HBNC+ home visits*

Indicator	Treatment			Control			Impact estimate (SE)
**Baseline**	**Endline**	**Endline-baseline**	**Baseline**	**Endline**	**Endline-baseline**
Ever received a visit (%)	44.1	68.7	24.6†	42.3	54.9	12.6†	10.48† (3.82)
Mean number of visits	1.6	2.6	1	1.4	1.8	0.5	0.54† (0.18)
Received the full schedule of visits (%)	18.8	39.1	20.4†	15.5	25.5	10†	9.41† (3.35)
N	953	1055		981	1100		
Mean number of visits conditional on being visited	3.5	3.8	0.3	3.3	3.3	0.1	0.21 (0.24)
N	420	725		415	604		

*Unweighted estimates reported. Cluster-adjusted standard errors (SE) are reported in parentheses. Impact estimates use diff-in-diff specification and control for a vector of child, mother and household characteristics, and state fixed effects.

†Significant at 1%.

The qualitative data suggests that low and delayed incentives were a driver of relatively low coverage compared to protocols and targets. Compared to incentives received from other programmes, such as institutional delivery and immunisation, most of the ASHAs reported that they were not satisfied with the low incentives received under HBNC+. As an ASHA from Madhya Pradesh said in an IDI: ‘No, I am not satisfied with the incentives, it is very less as compared to my workload’. Another ASHA from Bihar also said in an IDI: ‘Yes, it affects. If we will get good incentive, then only we would be happy in doing our work otherwise I don’t feel like working’. Some ASHAs said that they have expenses, such as travel expenses and mobile phone expenses, which were not reimbursed: ‘we have to bear our mobile expenses and conveyance’ (ASHA in an IDI, Madhya Pradesh). They also said that they did not receive incentives on time in their bank account. Complex incentive delivery systems also led to confusion over what incentives were received for which service. As stated by an ASHA from Bihar in an IDI: ‘After completing four follow up visits we get the form ready and submit it. I am eligible for incentives. We face a lot of challenges. We do not understand for which service we are getting the incentives’.

**Figure 2:**
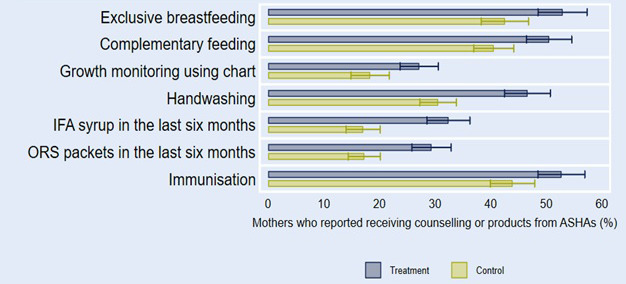
First stage: Content of home visits at endline.

In addition to the increase in the coverage of home visits, the programme also had an impact on the content of these visits. The home visits in the treatment areas were substantially more likely to adhere to HBNC+ protocol than the home visits in the control areas at endline. As shown in **Figure 2**, a significantly higher proportion of mothers reported receiving counselling on each HBNC+ component, and received supplies such as IFA and ORS packets, during home visits in the treatment districts than in the control districts.

### Outcomes

At the outcomes level, the impact evaluation investigated the impact of HBNC+ visits on eight key outcome indicators using the diff-in-diff methodology. As shown in **Table 2**, we do not find any evidence of statistically significant treatment effects on the key outcomes. Based on estimated 95% confidence intervals, however, we cannot rule out increases as large as 17 p.p. in the case of exclusive breastfeeding and 6.7 p.p. in the case of growth monitoring.

**Table 2:** Impact of the programme on key outcomes*

Indicator	Treatment			Control			Impact estimate (SE)
**Baseline**	**Endline**	**Endline-baseline**	**Baseline**	**Endline**	**Endline-baseline**
Children 3–5 mo of age exclusively fed breastmilk (%)	36.7	74.5	37.8†	43.6	76.1	32.5†	5.49 (5.85)
N	365	329		367	343		
Minimum diet diversity (%)	17.5	8.3	-9.2†	14.9	7.7	-7.2†	-1.38 (2.69)
N	675	724		679	751		
Mothers who washed their hands at three or more critical moments (%)	99.9	99.6	-0.3‡	99.9	99.7	-0.2	-0.13 (0.21)
N	1948	1985		1987	2064		
Growth monitored at least once in three months (%)	62.7	82.7	20.0†	59.1	77	17.9†	0.41 (3.21)
N	1455	1628		1457	1710		
Children 12–23 mo who received full immunisation (%)	51.9	47.9	-4.0	49.4	44.4	-5.0‡	-0.77 (3.63)
N	969	986		1041	993		
Mothers who play with their child (%)	60.4	81.1	20.7†	58.4	81.0	22.6†	-2.83 (2.78)
N	1948	1985		1987	2064		
Consumed IFA syrup twice per week in the last two weeks (%)		5.0			2.1		2.8†
N		1656			1721		
Treated with ORS (%)	57.4	44.7	-12.7				
N	47	76					

*Unweighted estimates reported. Cluster-adjusted standard errors (SE) are reported in parentheses. Impact estimates use diff-in-diff specification and control for a vector of child, mother and household characteristics, and state fixed effects, except for the indicators are IFA consumption (endline-only comparison) and treatment with ORS (pre-post in treatment areas only).

†Significant at 1%.

‡Significant at 5%.

However, the comparison of the endline values for IFA consumption shows a statistically significant difference, though this cannot be attributed to HBNC+ because of the lack of a counterfactual.

The qualitative study explored several reasons behind this lack of improvement in outcomes. Some related to system level constraints, including a lack of coordination with and reliance on other health providers (for example, growth monitoring is undertaken not by ASHAs but by village nutrition workers called Anganwadi Workers) and a lack of supplies. Nearly half of ASHAs in treatment areas reported having no stock of IFA syrup at endline, and a quarter reported having no stock of ORS.

In some areas, limited ASHA knowledge may have contributed to poor outcomes. At endline in treatment areas, ASHAs were found to have high levels of knowledge about some HBNC+ components (such as 98% of ASHAs knowing that exclusive breastfeeding should continue for six months), however for other components this was much lower (such as 65% of ASHAs knowing that growth monitoring should be done at least once a quarter, and 55% being aware of at least three critical moments for handwashing).

Beneficiaries and ASHAs also cited practical, community-level challenges as a reason for not adhering to the behaviours promoted. For example, some mothers could not exclusively breastfeed because they could not produce milk, or because they were not the sole caregiver. As an ASHA from Rajasthan stated in an IDI: ‘Some mothers do follow but not completely. Sometime when they go to field then their mother-in-law gives water and other things to the child’.

## DISCUSSION

The findings demonstrate that whilst there was a statistically significant increase in the number of home visits received by mothers because of the intervention, and that the content of these visits became more aligned with HBNC+ protocol in treatment districts compared to control areas, effective coverage remained low. This finding matches closely to the figures reported by the implementing team in their 2015 Annual Report [22] where 66% of children were reported to have received at least one visit (estimated using government Mother and Child Tracking System data) and 34% all four visits. These two data sources would have covered the same time period due to the retrospective nature of the evaluation survey and hence, this triangulation gives confidence to the coverage estimates.

However, the evaluation demonstrates no observed impact on key outcomes. This has also been documented elsewhere. A rapid assessment of HBNC+ [23] in Rajasthan found negligible or no differences in outcome levels between treatment and matched control areas in rates of exclusive breastfeeding, complementary feeding, immunisation, and handwashing. It also found a statistically significant effect on IFA consumption. These results successfully triangulate with the impact evaluation findings above and give confidence in the robustness of the findings.

However, this evaluation has its own limitations. The evaluation is unable to assess the impact on infant mortality, as this would have required a much larger sample to detect any meaningful impact and a longer evaluation period. A second limitation of the methodological approach is that the quasi-experimental estimation requires the parallel trends assumption to be upheld. As outlined in the methodology, the PSU surveys and key informant interviews detected no confounding factors that affected the treatment and control areas differently. This gives confidence to the evaluation findings. However, we are not able to reliably test for the parallel trends assumption. The results point to other drivers of change occurring in both treatment and control areas. For example, growth monitoring improved in all areas potentially either due to strengthening of the Anganwadi programme or pan-state behavioural change communication interventions. Hence, attributing changes to some of the outcomes is not possible and also beyond the scope of the evaluation.

It is also not possible to test robustly whether the lack of demonstrated effectiveness is due to low underlying efficacy of home visits (ie, home visits do not impact on care-seeking and healthy behaviours) or their low effective coverage (the intervention has an impact on those who receive it, but not enough people received it to detect changes at a population level). Further research would be required to assess the underlying efficacy of these home visits and identify opportunities for strengthening them. In other contexts, home visits by ASHAs have been shown to be efficacious at influencing behaviour and care-seeking, including in the neonatal period [12,24]. However, they have also been documented to be perfunctory and impersonalised due to low levels of trust between ASHAs and the community [25,26]. Evaluation of these CHW programmes would require perceptions of community members towards ASHAs providing these services to be established.

The evaluation suggests that achieving high effective coverage at scale remains a challenge. Even at endline in the treatment areas, only 39% of children had received the programme target of four visits, despite the additional training and incentives provided under HBNC+. This is consistent with the experience of the original HBNC programme for the neonatal period. Whilst this evaluation did not assess HBNC, the surveys measured its coverage. The baseline survey showed that, despite several years of HBNC implementation, only 7% of children were receiving at least six visits after institutional delivery, and only 4% seven visits after home delivery [21].

The baseline survey also identified issues that undermined the effectiveness of HBNC similar to those described in this evaluation of HBNC+. There were low levels of knowledge among ASHAs of the HBNC protocol, limited coverage of content during these home visits, low levels of referrals by ASHAs of children exhibiting danger signs, and low levels of patient satisfaction [21]. Explanatory reasons included the limited confidence of ASHAs in identifying danger signs, delayed incentives, and competing workload priorities. A broader review of HBNC showed that only 14% of ASHAs countrywide had received all rounds of training, training was of low quality and overly knowledge-based (rather than competency-based), incentives were delayed, supply chains (eg, for the ASHA drug kit) were weak, and supervision was limited [27]. Further studies have documented poor knowledge and skills among ASHAs in the provision of HBNC [28].

The systemic challenges identified that undermined the effectiveness of HBNC+ – a lack of supplies and commodities, heavy workloads, low and delayed compensation, and weak training systems – mirror the most pressing challenges facing CHW programmes globally [2,4]. It is increasingly understood that the binding constraints on CHW performance often lie outside the control of the CHW [29]. This reinforces the need for programme designs to consider the practical and organisational implications of new CHW activities with regards to training requirements, health system support, incentives, work location, and workload. The design of HBNC+ may have benefited from better attention to these systemic issues, particularly given the experience of HBNC. Whilst the focus of HBNC+ was to improve maternal and child health outcomes through home visits, further research would be needed to evaluate how these interventions can be adequately supported by more general, complementary, system strengthening activities. Engaging ASHAs more in the design process may also help increase the sensitivity of interventions to their needs and systemic predicaments [30].

Given that HBYC has already been scaled nationally, it will be important to identify ways to build functional systems and appropriately support ASHAs in attaining high coverage and quality and having maximum impact. Similar conclusions have been made in the face of the frequently disappointing results from community case management approaches to treating diarrhoea, pneumonia and malaria [31,32]. For HBYC, aside from system level strengthening, interventions will also need to target the demand side and socio-cultural barriers in adopting key health behaviours. For example, where neonatal home visits alongside other interventions to promote care seeking for pneumonia and diarrhoea have been effective in India, programmes have invested in additional demand side activities and community awareness along with strengthening drug availability and supervision [12,33,34]. There is also a need to build responsive and real-time monitoring and evaluation systems and conduct concurrent implementation research to achieve effectiveness at scale.

For HYBC to be successful at scale, a revision to the approach to delivering appropriate and timely incentives is likely to be required. The constraining impact of ineffective incentives on home visits is well established in the literature [35]. In India, delayed and low incentives have been shown to undermine intrinsic motivations of ASHAs [36]. A realistic examination of workload may also be required – HBNC+ is just one of 39 incentivised tasks that ASHAs are mandated to carry out. Rationalisation with other approaches to improving community health outcomes, including group based participatory learning and action approaches which have been shown to have high impact in India, may need to be considered [37]. Group based approaches have been shown to be as effective at promoting ECCD outcomes in India as home visits, for example, at under a third of the cost [38].

Beyond HBYC, whilst this evaluation provides lessons specific to a particular set of behaviours and services promoted through a specific schedule of home visits in India, the lessons on challenges to scale up of home visits by CHWs through government systems are generalized findings. The evaluation findings reinforce the need to focus not just on the intervention package but also on the broader system-level factors, particularly supply chains, supportive supervision, workload and incentives, as well as the demand-side factors, required to create an enabling environment for high coverage and quality and hence, achieve greater impact.

## CONCLUSION

The provision of training and incentives to ASHAs to deliver at least four visits to infants aged from three to 12 months led to a significant increase in the number of mothers receiving home visits. These home visits were also reported to be more closely following the HBNC+ protocols as measured by the reporting of different counselling messages or supplies by the mothers. However, only 39% of the women reported receiving the full schedule of HBNC+ visits, and there was no detectable effect of HBNC+ on average levels of outcomes. As HBNC+ has now been scaled nationally as HBYC, it will be important to reconsider current design elements (such as the incentive schedule) and solve the underlying demand-side constraints and systemic challenges such as workload and supply chains that will enable ASHAs to deliver HBYC at high coverage and quality at scale. Learning how to do so will require intensive concurrent monitoring, learning and evaluation. More generally, this paper contributes to the lessons on the challenges of achieving high effective coverage and quality of home visits by CHWs at scale, particularly in the post-neonatal period.
